# Depression and anxiety symptoms are related to pain and frailty but not cognition or delirium in older surgical patients

**DOI:** 10.1002/brb3.2164

**Published:** 2021-05-05

**Authors:** Sophia Wang, Brielle Cardieri, Hung Mo Lin, Xiaoyu Liu, Mary Sano, Stacie G. Deiner

**Affiliations:** ^1^ Department of Psychiatry Indiana University School of Medicine Indiana Alzheimer’s Disease Research Center Indianapolis IN USA; ^2^ Medical Education Program Icahn School of Medicine at Mount Sinai New York NY USA; ^3^ Department of Population Health Science and Policy Icahn School of Medicine at Mount Sinai New York NY USA; ^4^ Department of Anesthesiology Icahn School of Medicine at Mount Sinai New York NY USA; ^5^ James J. Peters VA Medical Center New York NY USA; ^6^ Department of Psychiatry Alzheimer's Disease Research Center Icahn School of Medicine at Mount Sinai New York NY USA; ^7^ Department of Anesthesiology Dartmouth Hitchcock Medical Center Lebanon NH USA

**Keywords:** aging, anxiety, depression, frailty, pain, postoperative cognitive decline, postoperative complications

## Abstract

**Objective:**

In community dwelling older adults, depression and anxiety symptoms can be associated with early cognitive decline. Symptoms of depression and anxiety are common in older adults prior to surgery. However, their significance is unknown. Our objective was to determine whether preoperative depression and anxiety symptoms are associated with postoperative cognitive decline (POCD) and in‐hospital delirium, in older surgical patients.

**Methods:**

We conducted a secondary data analysis of postoperative cognitive dysfunction in a cohort study of patients 65 and older undergoing elective noncardiac surgery. We used the Hospital Anxiety and Depression Scale (HADS) to screen for depression and anxiety symptoms at a home visit prior to surgery and 3 months after surgery. Patients with a history of psychiatric (major depressive disorder, bipolar disorder, and schizophrenia) or neurologic disorder (Parkinson's disease and stroke) were excluded from the parent study.

**Results:**

Out of the 167 patients, 9.6% (*n* = 16) reported significant depressive symptoms and 21.6% (*n* = 36) reported significant anxiety symptoms on preoperative screening. There was no association between preoperative or new‐onset postoperative depression and anxiety symptoms and the incidence of delirium or POCD three months after surgery. Patients with preoperative depressive symptoms had higher preoperative pain (scores 69 vs. 35.7, *p* = .002) and frailty (56 vs. 14.6, *p* <.001).

**Conclusion:**

In our cohort, we did not detect an association between preoperative depression and anxiety symptoms and neurocognitive disorders. Preoperative depression and anxiety symptoms were related to physical pain and frailty. Taken together, these suggest that in patients without a formal psychiatric diagnosis, preoperative depression and anxiety symptoms are related to physical state rather than a harbinger of early cognitive decline. Future studies are needed to understand the nature of the relationship between depression and anxiety symptoms and physical state in surgical patients.

## Graphical abstract

Preoperative mood symptoms were related to physical pain and frailty. Taken together, these suggest that in patients without a formal psychiatric diagnosis, preoperative mood symptoms are related to physical state rather than a harbinger of early cognitive decline. Future studies are needed to understand the nature of the relationship between mood symptoms and physical state in surgical patients.

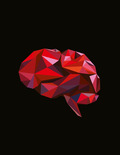

## 1 INTRODUCTION

Symptoms of depression and anxiety are common in older surgical patients. (Sorel et al., 2019; Strøm et al., 2018) There are many reasons for these symptoms in the context of upcoming surgery including psychological stress, concurrent medical illness, and pain. However, depression and anxiety in older adults could also be a sign of incipient neurodegenerative disease. In older community dwelling patients, depression and anxiety symptoms are each associated with transition from mild cognitive impairment (MCI) to Alzheimer's disease (AD). (Becker et al., 2018; Byers & Yaffe, 2011; Gulpers et al., 2016; Morimoto et al., 2014; Schuurmans & Balkom, 2011; Wang & Blazer, 2015) In older surgical patients, neurocognitive disorders, namely delirium and postoperative cognitive decline (POCD), are common postoperative complications, affecting 10%–60% and 10%–15% of older surgical patients, respectively. (Brown & Purdon, 2013; Paredes et al., 2016)

While POCD and dementia are not analogous, patients with early and often unrecognized dementia are higher risk for postoperative cognitive complications. The relationship between preoperative depression and anxiety and POCD is not well understood. Most preexisting cognitive impairment is mild, and in many cases, not evident to the patient or provider. Depression and anxiety symptoms, assessed with brief standardized instruments could represent a practical way to screen for a risk factor for cognitive impairment and decline. Similarly, there is evidence that preoperative depression is associated with the development of delirium, which has also been associated with a higher risk of POCD.

To test whether preoperative depression and anxiety are associated with cognitive complications in older surgical patients, we performed a secondary analysis of a prospective cohort study PRESERVE (Optimizing Postoperative Cognitive Dysfunction in the Elderly). We hypothesized that preoperative depression and anxiety symptoms will be associated with the development of postoperative delirium. Secondarily, we investigated whether patients with new or worsening depression and anxiety symptoms after surgery developed POCD and planned to adjust for known confounding factors including pain and frailty.

## 2 METHODS

Our study is a secondary analysis of PRESERVE, a prospective observational cohort study. Patients were enrolled from October 2016 to November 2019. Patients who were 65 years or older and underwent major noncardiac elective surgery at The Mount Sinai Medical Center, a tertiary academic hospital in New York City, were eligible to participate. Major surgery was defined as a hospital stay of at least 2 or more days. Exclusion criteria were preoperative delirium or dementia based on participant report and medical chart review. Patients with mild cognitive impairment (MCI) were included, but patients with history of stroke and Parkinson's disease, and psychosis or recent psychiatric hospitalization for any reason), cardiac or intracranial surgery, lack of capacity to consent to participate in research, or emergency surgery were excluded. All subjects signed written informed consent. The study was approved by the Mount Sinai Institutional Review Board and registered with Clinical Trials Gov (NCT 02650687). The primary outcome of the parent study was to determine the relationship between cognitive decline and physical recovery.

### 2.1 Baseline clinical characteristics

Patients completed a preoperative evaluation 30 days to 24 hr before their elective surgery. At the preoperative evaluation, patients provided sociodemographic data, information about medical and psychiatric disorders, and medication lists. A board‐certified anesthesiologist determined their American Society of Anesthesiologists (ASA) Physical Status Classification System on a scale of I (normal, healthy patient) to VI (brain dead). Frailty was determined via the FRAIL Scale Questionnaire administered before surgery. (Morley et al., 2012) Sleep latency and duration were recorded using the Pittsburgh Sleep Quality Index (PSQI). (Buysse et al., 1989) We included sleep because there is evidence to suggest that sleep may mediate the relationship between frailty and cognition. (Ensrud et al., 2009; Kaur et al., 2019) They also completed preoperative self‐assessments of their depression and anxiety using the Hospital Anxiety and Depression Scale (HADS), (Zigmond & Snaith, 1983) and pain (Geriatric Pain Scale, GPS) (Ferrell et al., 2000) and a neuropsychological battery (ADRC UDS). (Weintraub et al., 2009) (See below for additional details.)

### 2.2 Intraoperative characteristics

Patients underwent major noncardiac surgery (general, spinal, thoracic, or urologic) with general anesthesia. Anesthesiologists received minimal directions regarding anesthetic choice, as previous literature has not shown a difference between classes of anesthetics (*e.g*., total intravenous anesthesia and gas anesthetics) and postoperative cognitive outcomes. However, they were asked to avoid using midazolam, nitrous oxide, ketamine, or etomidate, as the primary outcome requires the analysis of a processed intraoperative EEG, and these drugs cannot be accounted for in the device's proprietary algorithm. Intraoperative data, including blood pressure, anesthetics and narcotics administered, surgical and anesthesia duration, were recorded.

### 2.3 Delirium assessment

Patients were extubated at the end of surgery and transferred to the postanesthesia care unit (PACU) and then to the surgical wards. Trained research staff screened for delirium once in the PACU and twice daily (morning and evening) using the Confusion Assessment Method (CAM) ICU (Inouye et al., 1990) until hospital discharge.

### 2.4 Self‐assessment scales

Patients completed self‐assessment scales at the preoperative evaluation and the 3 month follow‐up visit. To measure depression and anxiety symptoms, patients completed the Hospital Anxiety and Depression Scale (HADS), which is comprised of a depression and anxiety subscale, with 7 items each. Each item is rated on a four point (0–3) scale, with possible subscale scores ranging from 0 to 21. (Zigmond & Snaith, 1983) Cutoff of ≥8 was used to denote the presence of “likely depression or anxiety”. For the assessment of pain, patients completed the Geriatric Pain Measure, a 24‐item questionnaire that is easy to administer and has significant validity and reliability in older persons with multiple medical problems. (Ferrell et al., 2000) For the assessment of sleep, patients completed the PSQI, a 9‐item questionnaire measures the quality and patterns of sleep in the older adult. (Buysse et al., 1989) For the assessment of frailty, patients completed the FRAIL Scale (Morley) which asks patients to complete questions about fatigue, resistance, aerobic endurance, number of medical illnesses, and recent significant weight loss. (Ferrell et al., 2000).

### 2.5 Cognitive assessment

All patients were assessed with a full neuropsychological battery prior to surgery at the same time the self‐assessment scales administered (within 30 days but at least 24 hr prior) and again at 3 months after surgery. We used the California Verbal Learning Test (CVLT‐II) and the Uniform Dataset Battery (UDS) from the Alzheimer's Disease Research Center. (Weintraub et al., 2009) UDS tests include Trail Making Test, subtests from the Wechsler Adult Intelligence Scale (WAIS‐R Digit Symbol and WAIS‐III Digit Span), Logical Memory Story A, Immediate and Delayed Recall, Animal and Vegetable verbal fluency, Boston Naming Test, and the Mini‐Mental Status Exam (MMSE). Executive function was tested with the Trail Making Test, WAIS‐R Digit Symbol, WAIS‐III Digit Span. Memory and language were assessed with category fluency (Animal and Vegetable verbal fluency), the CVLT‐II, and Logical Memory (Immediate and Delayed Recall) and Boston Naming Test. Neuropsychological testing was performed by trained personnel.

### 2.6 Statistical analyses

The sample size calculation for the protocol was based on the primary outcome for the parent grant, instrumental activities of daily living, as detailed in Deiner et al. (2020) Sociodemographic, clinical characteristics, intraoperative characteristics, and medication data were compared using parametric (Chi–square, Fisher's exact) or nonparametric (Mann–Whitney) tests. Continuous variables are presented as mean ±standard deviation (*SD*) or median [Interquartile range] and categorical variables as count (%).

### 2.7 Identification of cognitive decline

For each cognitive test, the individual scores obtained at baseline and 3 months after surgery were normalized to create *z*‐scores (the difference between the individual score and the baseline average was divided by the baseline standard deviation). Prior to normalization, logarithmic transformation was performed for Trails A and B and the sign corrected so that when change scores were created, higher values were always “better.” The average of the normalized baseline *z* scores across all individual tests was then used to indicate the overall baseline cognitive performance. We reported a composite score which was the sum of the *z*‐scores of all tests. Group comparison of changes in cognitive score from baseline to 3 months was adjusted for their respective baseline score using least squares regression.

We calculated the change in *z*‐score from baseline to 3 months. The effect of learning from repetitive testing (practice effect) was calculated as the average of the change scores across all individuals. The difference between an individual's change score combined with the practice effect was compared to the average score of our population at baseline. Patients with a decline of 1 or more *SD* were defined as having cognitive decline. Note that the threshold is similar to prior study, but is not the method of Evered *et al* which requires knowledge of a subjective complaint and functional status. (Evered et al., 2018) We chose to use our own patients as the normative group because they were lower scoring prior to surgery on almost every test at baseline relative to age normative data. This was true despite the fact that our patients were overall highly educated. Thus, it was not possible to calculate a reliable change index (RCI) to adjust for practice effects across testing.

Cluster analysis was used to perform grouping of the cognitive tests. The variable cluster analysis (VARCLUS) procedure in SAS (v 9.4) was implemented to find clusters of cognitive tests that were correlated as possible within the group and not with tests in other clusters. The algorithm was binary and divisive, i.e., at the beginning of the analysis all cognitive tests start in one cluster and splitting continues until a stopping criterion (based on eigenvalues) is reached.

## 3 RESULTS

**FIGURE 1:**
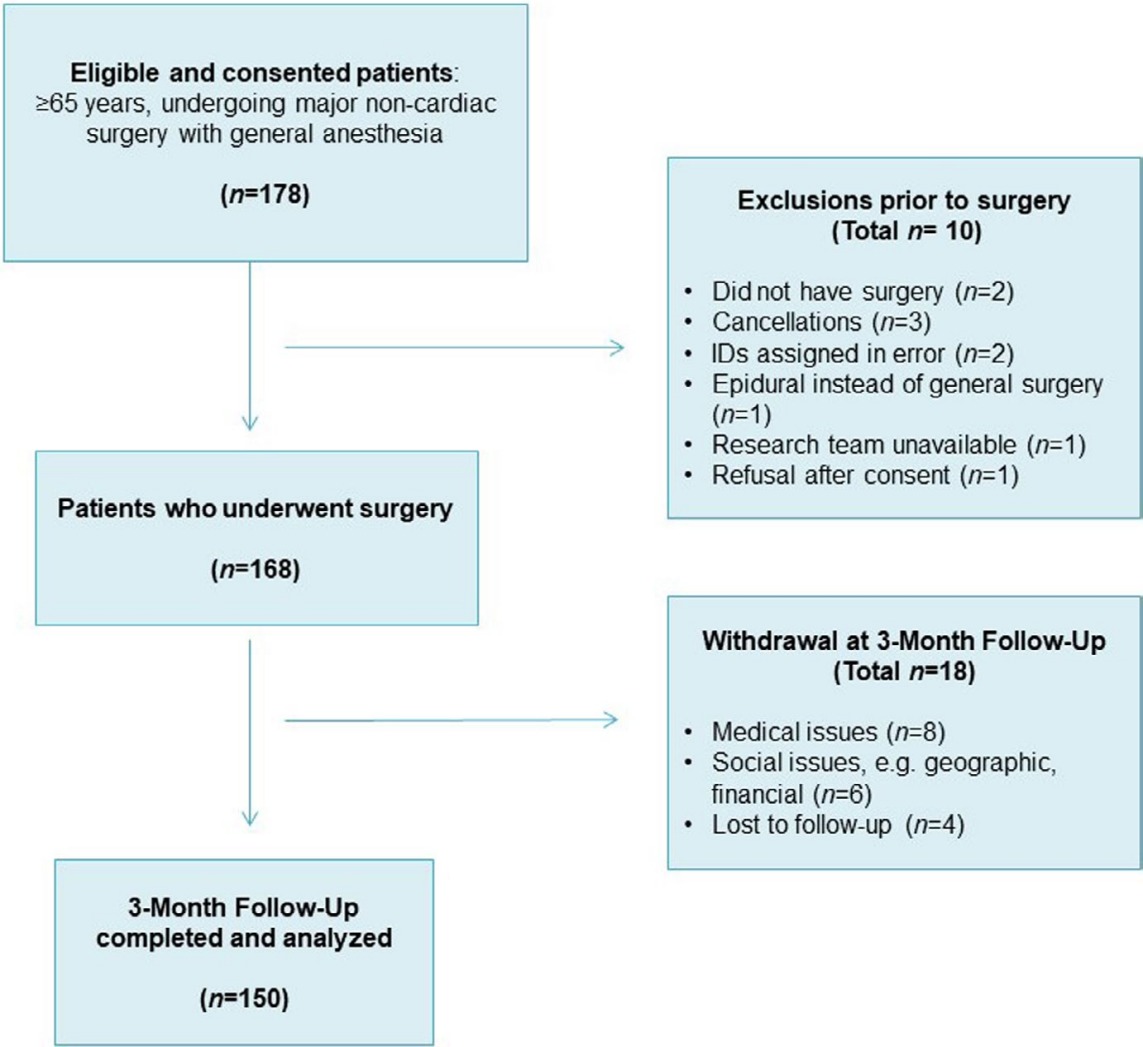
Strengthening the reporting of observational studies in epidemiology (STROBE) diagram

A total of 178 patients were eligible and signed consent, 167 patients underwent surgery. A total of 149 subjects successfully completed the 3 months follow‐up. Figure 1 depicts the flow diagram of subject recruitment and withdrawal.

Out of these 167 patients, 16/167 (6%) screened in for likely depression (as defined by a HADS depression score of ≥8) prior to surgery, and 36/167 (21.6%) screened in for preoperative anxiety. Table 1 compares the baseline clinical and preoperative characteristics for those with and without likely depression and anxiety. Patients who screened for depression had fewer years of education (14 [12, 16] v. 16 [13, 18], *p* <.001). There was no difference in depression scores by surgery type, but a higher proportion of patients had anxiety prior to spine surgery. At baseline, patients with likely depression reported higher pain scores (69 vs. 35.7, *p* =.002). Frailty was significantly greater among patients with likely depression (56% vs. 14.6%, *p* <.001) and anxiety (30.6% vs. 15.3%, *p* <.037).

**TABLE 1:** Demographics

	*n* ^a^	Total *n* = 167	Preop symptoms of depression			Preop symptoms of anxiety		
			Yes (*n* = 16)	No (*n* = 151)	*p*‐val^b^	Yes (*n* = 36)	No (*n* = 131)	*p*‐val^c^
Age (years)	167	70 [67,74]	68 [66,74.5]	70 [67,74]	0.347	70 [66,73.5]	70 [67,75]	0.280
Education (years)	167	16 [13,18]	14 [12,16]	16 [13,19]	0.014	15.5 [12.5,18]	16 [13,19]	0.507
Race	166				<0.001			0.337
Black		29 (17.5)	1 (6.3)	28 (18.7)		5 (14.3)	24 (18.3)	
White Hispanic		12 (7.2)	6 (37.5)	6 (4)		5 (14.3)	7 (5.3)	
White non‐Hispanic		124 (74.7)	9 (56.3)	115 (76.7)		25 (71.4)	99 (75.6)	
Asian		1 (0.6)	0	1 (0.7)		0	1 (0.8)	
Gender	167				0.922			0.231
Male		75 (44.9)	7 (43.7)	68 (45.0)		13 (36.1)	62 (47.3)	
Female		92 (55.1)	9 (56.3)	83 (55.0)		23 (63.9)	69 (52.7)	
Pain score	167	38.1[11.9,66.6]	69.0[44.0,82.1]	35.7 [7.1, 61.9]	0.002	55.9 [34.5,76.2]	33.3[7.1,61.9]	0.003
Frail	167	31 (18.6)	9 (56.3)	22 (14.6)	<0.001	11 (30.6)	20 (15.3)	0.037
Not frail		136 (81.4)	7 (43.7)	129 (85.4)		25 (69.4)	111 (84.7)	
ASA status	167				0.822			0.880
II		57 (34.1)	5 (31.3)	52 (34.4)		11 (30.6)	46 (35.1)	
III		104 (62.3)	10 (62.5)	94 (62.3)		24 (66.7)	80 (61.1)	
IV		6 (3.6)	1 (6.3)	5 (3.3)		1 (2.8)	5 (3.8)	
Surgery type	167				0.090			0.040
Spine		71 (42.5)	9 (56.3)	62 (41.1)		21 (58.3)	50 (38.2)	
Thoracic		19 (11.4)	4 (25)	15 (9.9)		6 (16.7)	13 (9.9)	
Urologic		28 (16.8)	1 (6.3)	27 (17.9)		3 (8.3)	25 (19.1)	
General		49 (29.3)	2 (12.5)	47 (31.1)		6 (16.7)	43 (32.8)	
Baseline *z*‐score	167	0.06 [−0.3, 0.4]	0.02 [−0.4, 0.2]	0.06 [−0.3, 0.5]	0.321	0.02 [−0.3, 0.2]	0.06 [−0.3, 0.5]	0.798
MMSE	167	29 [28,30]	28 [27,29.5]	29 [28,30]	0.118	29 [27,30]	29 [28,30]	0.883
History Depression	167	23 (13.8)	6 (37.5)	17 (11.3)	0.011	9 (25)	14 (10.7)	0.052
No depression		144 (86.2)	10 (62.5)	134 (88.7)		27 (75)	117 (89.3)	
History of Anxiety	167	31 (18.6)	7 (43.7)	24 (15.9)	0.013	16 (44.4)	15 (11.5)	<0.001
No anxiety		136 (81.4)	9 (56.3)	127 (84.1)		20 (55.6)	116 (88.5)	
Sleep duration	166	8.0 [7,8.5]	8.1 [5.5,8.8]	7.8 [7,8.5]	0.856	7.5 [6.0,8.5]	8 [7,8.8]	0.297
Sleep latency >30 min	167	61 (36.5)	7 (43.7)	54 (35.8)	0.528	20 (55.6)	41 (31.3)	0.007
Sleep latency <30 min		106 (63.5)	9 (56.3)	97 (64.2)		16 (44.4)	90 (68.7)	
Preoperative medication								
Sleep med	167	13 (7.8)	1 (6.3)	12 (8.0)	1.000	4 (11.1)	9 (6.9)	0.481
Benzodiazepines	167	28 (16.8)	5 (31.3)	23 (15.2)	0.150	10 (27.8)	18 (13.7)	0.046
Antidepressants	167	39 (23.4)	7 (43.8)	32 (21.2)	0.060	13 (36.1)	26 (19.9)	0.041
Preop opioid	167	20 (12.0)	4 (25)	16 (10.6)	0.105	9 (25)	11 (8.4)	0.016
Polypharmacy	167	73 (43.7)	9 (56.3)	64 (42.4)	0.288	19 (52.8)	54 (41.2)	0.216

^a^ The number of patients with available data.

^b^ For both comparisons between preoperative symptoms of depression Yes versus No, Chi‐square test was used for gender (with *df*=1), ASA status (2), polypharmacy (1), Fisher's exact test was used for race, frail, surgery category, history of depression, history of anxiety, sleep med, benzodiazepines, antidepressants, baseline opioid, and Mann–Whitney *U* test with *df* = 165 was used for continuous variables, as appropriate.

^c^ For both comparisons between preoperative symptoms of Anxiety Yes versus no, Chi‐square test was used for gender (*df* = 1), frail (1), surgery category (3), benzodiazepines (1), antidepressants (1), polypharmacy (1), Fisher's exact test was used for race, ASA status, history of depression, history of anxiety, sleep med, baseline opioid, and Wilcoxon rank sum test with *df* = 165 was used for continuous variables, as appropriate.

Use of sleep medications and benzodiazepines was common in this group, especially considering that most are Beers criteria medications contraindicated for older adults. 13/167 (7.8%) used sleep medications, and 28/167 (16.8%) used benzodiazepines. (American Geriatrics Society, 2019) Antidepressant use was also common; 39/167 (23.4%) patients which was more common in patients with depression and anxiety symptoms. 20/167 (20%) used opioids before surgery, which seems small given the amount of pain reported; this was not different between patients with and without depression, but was statistically significantly different between patients with and without anxiety. Polypharmacy was common, 73/167 (43%) were on 5 or more medications, which was not different between patients with and without depression and anxiety symptoms.

We analyzed our cohort for differences for 3 cognitive domains (executive function, memory and language, and episodic memory) and a composite score based on the neuropsychological battery (Table 2) (all *p* >.05). Table 3 demonstrates the relationship of persistent or new depression and anxiety to cognitive decline, delirium, sleep, and surgical type. At the three month follow‐up, there was no difference in the incidence of POCD between patients who did and did not experience an improvement in depression or had persistent depression (Table 3). Similarly, there was no difference in the incidence of POCD in patients who did and did not experience an improvement in anxiety. 7/16 patients with preoperative depressive symptoms (43.7%) developed delirium compared to 33/151 (21.8%) *p* =.065. 6/27 (22.2%) of patients with anxiety developed delirium compared to 25/93 (26.9%) *p* =.63. 40% of patients who had persistent or new depression symptoms had postoperative delirium compared to 23.5% of patients who improved or had no depression but this was not significant (*p* =.140).

**TABLE 2:** Cognitive outcomes by preoperative depression and anxiety

	Total	D + *n* = 15	D‐ *n* = 135	*p*‐val	A + *n* = 34	A‐ *n* = 116	*p*‐val
Cog decline	21 (14)	2 (13.3)	19 (14.1)	1.000^a^	5 (14.7)	16 (13.8)	1.000^a^
No decline^a^	129 (86)	13 (86.7)	116 (85.9)		29 (85.3)	100 (86.2)	
CVLT decline	24 (16)	2 (13.3)	22 (16.3)	1.000^a^	6 (17.6)	18 (15.5)	0.766^b^
No decline	126 (84)	13 (86.7)	113 (83.7)		28 (82.4)	98 (84.5)	
Mem lang decline	21 (14)	1 (6.7)	20 (14.8)	0.700^a^	6 (17.7)	15 (12.9)	0.574^a^
No Decline	129 (86)	14 (93.3)	115 (85.2)		28 (82.3)	101 (87.1)	
Exec function decline	21 (14)	2 (13.3)	19 (14.1)	1.000^a^	9 (26.5)	12 (10.3)	0.025^a^
No decline	129 (86)	13 (86.7)	116 (85.9)		25 (73.5)	104 (89.7)	

^a^ Fisher's exact test was used.

^b^ Chi‐square test with 1 *df* was used.

**TABLE 3:** Improvement and persistence of depression and anxiety by surgery type, cognition, delirium, pain, and sleep

	Depressive symptoms			Anxiety symptoms		
	Improves/none (*n* = 132)	New or persistent (*n* = 17)	*p*‐value	Improves/none (*n* = 129)	New or persistent (*n* = 20)	*p*‐value
Cog decline	18 (13.6)	3 (17.7)	0.710^a^	17 (13.2)	4 (20)	0.487^a^
No decline	114 (86.4)	14 (82.3)		112 (86.8)	16 (80)	
Postop delirium			0.140^a^			0.620^b^
No	101 (76.5)	10 (58.8)		97 (75.2)	14 (70)	
Yes	31 (23.5)	7 (41.2)		32 (24.8)	6 (30)	
Pain @ 3 M			0.002^b^			<0.001^b^
GPM score<30	82 (62.1)	4 (23.5)		84 (65.1)	2 (10)	
GPM score ≥30	50 (37.9)	13 (76.5)		45 (34.9)	18 (90)	
Sleep latency @ 3 M			0.128^b^			0.018^b^
No	87 (65.9)	8 (47.1)		87 (67.4)	8 (40)	
Yes	45 (34.1)	9 (52.9)		42 (32.6)	12 (60)	
Surgery type			0.510^a^			0.104^a^
Spine	57 (43.2)	8 (47.1)		53 (41.1)	12 (60)	
Thoracic	12 (9.1)	3 (17.7)		13 (10.1)	2 (10)	
Urologic	22 (16.7)	3 (17.7)		25 (19.4)	0	
General	41 (31.1)	3 (17.7)		38 (29.5)	6 (30)	

^a^ Fisher's exact test was used.

^b^ Chi‐square test with 1 *df* was used.

Persistent or new anxiety symptoms was not associated with a significant difference in postoperative delirium (*p* =.620) There was a trend toward spine surgery patients experiencing persistent anxiety but this was not statistically significant (*p* = 104).

## 4 DISCUSSION

In a cohort of older major noncardiac surgery patients, preoperative depression and anxiety symptoms were related to the presence of pain and frailty. We failed to detect an association between preoperative depression or anxiety symptoms and postoperative cognitive decline or delirium. We also did not find an association between worsening/new depression and anxiety after surgery was not related to postoperative cognition. Taken together, these suggest that in the older surgical patients, depression and anxiety symptoms are related to physical state rather than a risk factor for POCD.

Also, although participants did not undergo formal psychiatric evaluation, the reported prevalence of depression and anxiety symptoms in this cohort is consistent with previous epidemiologic studies examining the prevalence of depressive and anxiety disorders, although anxiety was slightly higher, possibly due to concerns about upcoming surgery. (Byers et al., 2010) Studies have found a relationship between severity of depression and anxiety symptoms and Alzheimer's disease. (Gracia‐García & de‐la‐Cámara et al., 2015; Mah et al., 2015) Patients with higher severity depression and anxiety symptoms may be a different population, and although less likely to be represented in this type of surgical cohort, potentially with a distinct set of needs and risk factors.

Many of the patients had long standing use of pain medications, antidepressants, and anxiolytic medications. The effects of this outpatient medication regimen could have had on depression and anxiety symptoms or cognition may underreported in the perioperative literature. It is likely that there is a complex relationship between depression and anxiety symptoms in the preoperative phase and in the course of surgical recovery and pain. Our study confirmed a relationship between preoperative depressive and anxiety symptoms with higher pain scores at baseline and 3 months after surgery. Moreover, persistent or worsening depressive and anxiety symptoms were associated with higher 3‐month pain scores. Earlier studies such as depression and anxiety have been shown to be related to higher postsurgical pain. (Pan et al., 2019) The relationship we found between depression and anxiety symptoms and pain after surgery points to an important area for improvement of patient centered outcomes with the potential to improve physical recovery.

The association between preoperative depression symptoms and frailty in our cohort is consistent with previous studies. (Mah et al., 2015) A recent meta‐analysis suggests there is relationship between depression symptoms and frailty and vice versa. (Soysal et al., 2017) We also found a relationship between preoperative anxiety and frailty. While there are not many studies on this topic, one study did find that anxiety is a risk factor for the development of frailty, and similarly, frailty is associated with anxiety. (Bernal‐López et al., 2012) However, one major challenge is the overlap of symptoms (such as fatigue and motivation) between frailty scales and DSM‐5 criteria, particularly for major depressive disorder and generalized anxiety disorder. Recent systematic reviews of frailty scales suggest that the psychometric properties of these scales are limited. (Sutton et al., 2019, 2016) Cognitive frailty is the co‐occurrence of mild cognitive deficits and physical frailty and also includes depression and anxiety symptoms in both medically ill and presurgical geriatric populations. Additional work is needed to examine whether the multidomain construct of cognitive frailty is a useful way to risk stratify postoperative older adults.

Our study was a secondary analysis of a prospective study, and we did not detect a clinically significant association between preoperative depressive symptoms and longer term cognitive recovery. Our findings are supported by an earlier study which found that preoperative depression and anxiety were not associated with changes in cognition before and after surgery. (Oyeyemi et al., 2020) Also, we found that the incidence of delirium in patients with preoperative depression was twice that of patients without preoperative depression. This is consistent with earlier studies found that preoperative depression was associated with postoperative delirium, (Eshmawey et al., 2019; Leung et al., 2005) although one study of cardiac surgical patients did not find any relationship between affective symptoms and postoperative delirium. (Detroyer et al., 2008) Further studies will be needed to determine whether preoperative depressive symptoms are a risk factor for postoperative delirium.

One limitation of this study is the use of a screening measure (HADS) primarily designed to rapidly screen for both depression and anxiety. The HADS was created to identify depressive and anxiety symptoms while minimizing overlap with somatic symptoms, which frequently occur in hospitalized patients. The HADS depression subscale focused on depressed mood and anhedonia. Although that the HADS is not based on the DSM‐IV TR criteria for major depressive disorder, it does perform comparably to the PHQ‐9 as a screening instrument for major depressive disorder. (Löwe et al., 2004; Stafford et al., 2007; Walker et al., 2007) Nevertheless, we cannot exclude that other depressive scales such as the Geriatric Depression Scale‐15 or 30 and Beck Depression Inventory could be predictive of POCD. We did not include a formal neuropsychiatric evaluation to confirm whether these symptoms met criteria for a DSM‐5 diagnosis, so our findings may not be generalizable to other scales or other populations.

Another limitation is the measurement of a single preoperative timepoint. Kaup et al showed that trajectories of depression, as compared to single time measurements of depression, may be more informative about incident dementia. (Kaup et al., 2016) We inquired but did not formally administer dementia evaluations. Capturing multiple timepoints of depression and anxiety symptoms during both the preoperative and postoperative time periods may be needed to more deeply understand the relationship between these symptoms and POCD. Some studies which primarily focused on depression and often are conducted over several years. (Byers & Yaffe, 2011) Future studies could include a later timepoint to better understand the trajectory of recovery from the immediate surgical trauma. It would also be important to clarify whether depression and anxiety disorders of greater severity, for example, a DSM‐5 diagnosis of major depressive disorder or generalized anxiety disorder, as compared to subsyndromal depressive and anxiety symptoms, will be associated with POCD.

## 5 CONCLUSION

Our study is one of only a few to examine the role of depression and anxiety symptoms and cognitive decline in a surgical cohort. We found that depression and anxiety symptoms were related to physical state: pain and frailty and not cognitive recovery after surgery. The relationship we found between depression and anxiety symptoms and pain after surgery points to an important area for improvement of patient centered outcomes with the potential to improve physical recovery. Additional work is needed to understand the directionality and magnitude of the association between physical frailty, recovery, pain, and depression and anxiety symptoms.

## CONFLICT OF INTEREST

Dr. Sophia Wang, Dr. Xiaoyu Liu, and Dr. Hung Mo Lin have nothing to declare. Dr. Mary Sano is a consultant for VTV, Hoffman‐LaRoche Ltd, Biogen Idec, CogRx, Brackett, Eisai Inc., Eli Lilly and Company, member of the DSMB Member for AZTherapies and for NIA “ASPREE”, and adjudicator for Trial Endpoint for Takeda Pharmaceutical Company Limited. Dr. Stacie Deiner has been an expert witness testimony and Merck consultant.

## AUTHOR CONTRIBUTIONS

SW contributed to manuscript drafting and edits; BC involved in data analysis, study design, and manuscript; HL and XL involved in data analysis and manuscript edits; MS involved in study design and manuscript edits; SD involved in study design, data analysis, and manuscript drafting and edits.

### PEER REVIEW

The peer review history for this article is available at https://publons.com/publon/10.1002/brb3.2164.
